# Association between tracking of extracurricular sports practice and weight status during childhood: a prospective cohort study

**DOI:** 10.1590/1516-3180.2020.0379.R1.18012021

**Published:** 2021-04-26

**Authors:** Vinícius Müller Reis Weber, Julio Cesar da Costa, Hélcio Rossi Gonçalves, Vitor Hugo Ramos Machado, Marcelo Romanzini, Enio Ricardo Vaz Ronque

**Affiliations:** I MSc. Physical Education Professional and Doctoral Student, Laboratory of Physical Activity and Health, Center for Physical Education and Sports, Universidade Estadual de Londrina (UEL), Londrina (PR), Brazil.; II MSc. Physical Education Professional and Doctoral Student, Laboratory of Physical Activity and Health, Center for Physical Education and Sports, Universidade Estadual de Londrina (UEL), Londrina (PR), Brazil.; III PhD. Physical Education Professional and Adjunct Professor, Sport Department, Center for Physical Education and Sports, Universidade Estadual de Londrina (UEL), Londrina (PR), Brazil.; IV MSc. Physical Education Professional, Doctoral Student and Professor, Department of Physical Education, Universidade Paranaense (UNIPAR), Umuarama (PR), Brazil.; V PhD. Physical Education Professional and Adjunct Professor, Laboratory of Physical Activity and Health, Center for Physical Education and Sports, Universidade Estadual de Londrina (UEL), Londrina (PR), Brazil.; VI PhD. Physical Education Professional and Associate Professor, Laboratory of Physical Activity and Health, Center for Physical Education and Sports, Universidade Estadual de Londrina (UEL), Londrina (PR), Brazil.

**Keywords:** Body mass index, Child, Overweight, Public health, Growth, Kindergarten, Weight control, Teenager

## Abstract

**BACKGROUND:**

Overweight and obesity have reached epidemic prevalences. Obesity control involves many factors and needs to begin early in childhood.

**OBJECTIVES:**

To ascertain the association between tracked extracurricular sports practice and weight status; and to analyze tracking of overweight and obesity among school-aged children.

**DESIGN AND SETTING:**

Prospective cohort study conducted in 13 public schools in Cianorte, Paraná, in 2012-2016.

**METHODS:**

The sample comprised 2459 schoolchildren in Cianorte, of mean age 6.3 years at baseline and 9.4 years at follow-up. Body mass index was calculated from body mass and height measurements. The children were grouped as normal weight, overweight or obese. Information on extracurricular sports practice was collected through the dichotomous question “Do you participate in any extracurricular sports?” (“yes” or “no”).

**RESULTS:**

Tracking of weight status showed that 75.5% maintained this, with kappa of 0.530. Tracking of extracurricular sports practice showed that 80.9% maintained this, with low concordance (kappa of 0.054). Weight status correlation between baseline and follow-up showed that overweight or obese individuals were 4.65 times (CI: 4.05-5.34) more likely to maintain the same classification or move from overweight to obese at follow-up. Correlation of extracurricular sports practice with overweight or obesity at follow-up was not significant.

**CONCLUSIONS:**

These results demonstrated that overweight or obese children were at higher risk of gaining weight than were normal-weight children. In addition, the proportion of these children who maintained extracurricular sports practices over the years was low. Maintenance of this variable was not associated with weight status.

## INTRODUCTION

Elevation of body mass index (BMI) levels has reached an alarming status within public health, both in developed and in developing countries. This has been seen as a gradual rise in the prevalence of overweight and obesity in different age groups.^1^^,^^2^^,^^3^^,^^4^^,^^5^ In a systematic review performed by Ng et al.,^6^ the prevalence of overweight or obesity was reported to be approximately 47% among children worldwide. Among the different factors that have contributed to this increase is a decline in physical activity levels.

In Brazil, it has been estimated that 269.6 million dollars are spent annually on combating obesity and related complications.^7^ A survey conducted in state capitals in 2014 showed that the prevalence of overweight in the Brazilian adult population was more than 51%.^8^ Among children and adolescents, the prevalence of overweight was 22% and 24%, respectively.^9^ More than 70% of subjects who presented higher adiposity in childhood remained under the same condition during adolescence.^10^ This is worrying, as the adolescent population presents a higher risk of maintaining this indicator in adulthood.^11^^,^^12^

The Bogalusa Heart Study, conducted between the 1970s and 1990s, indicated that obesity in children and adolescents is linked, respectively, to 2.4 and 7.1 times higher risk of presenting high cholesterol and triglycerides, in comparison with the population with normal body mass index.^12^ This increase in body mass index is associated with different risk factors for cardiovascular diseases, for example: high blood pressure, dyslipidemias, fasting insulin and risk score.^13^^,^^14^

Sports practice, predominantly performed in schools, has been considered to be one of the main forms of elevating daily energy expenditure among school-age children, and this is associated with weight control.^15^ However, little is known about the longitudinal impacts of sports practice on prevention of overweight in childhood, since the majority of studies have attempted to observe this occurrence using cross-sectional designs.^15^^,^^16^^,^^17^ Such studies do not longitudinally evaluate changes in or maintenance of weight status, or sports practice, in this population.

## OBJECTIVE

The aim of this study was to ascertain the association between tracked extracurricular sports practice and weight status; and to analyze the tracking of overweight and obesity among schoolchildren during childhood.

## METHODS

### Study design and sample selection

The data used in this study formed part of a database named: “Relationship of high blood pressure with body composition indicators among students in Cianorte, Paraná”. This project was carried out between 2012 and 2016 and was approved by a local university ethics committee for human research (no. 1,362,937). All the parents were informed of the procedures and only the children whose parents had signed a consent statement participated in the research. Data were collected by a previously trained researcher with understanding about protocols.

Cianorte is a municipality in the state of Paraná, Brazil, with 69,958 inhabitants, a literacy rate of 94.4%, human development index of 0.755 and *per capita* gross domestic product of 29,321 reais in 2015.

The sample was composed of all students enrolled in the municipality’s 13 public schools, aged 5-8 years. All students were invited to participate in the research, and only those who were absent on the day of the test were not included in the sample. For the present study, only the data from the students who underwent both baseline and follow-up measurements (follow-up three years later) were used in the analysis, totaling 2,459 subjects in the sample. The total number of children who dropped out from the research was 981 individuals. A comparison between the children who dropped out and those who completed the follow-up is presented in Table 1.

**Table 1.** Dropout analysis

	5 years old		6 years old		7 years old		8 years old	
	Dropped out (n = 115)	Followed (n = 525)	Dropped out (n = 172)	Followed (n = 881)	Dropped out (n = 170)	Followed (n = 764)	Dropped out (n = 524)	Followed (n = 289)
Age (years)	5.1 (4.9-5.3)	5.2 (5.0-5.3)	6.0 (5.8-6.2)	6.0 (5.7-6.3)	7.0 (6.8-7.3)	7.0 (6.7-7.2)	**8.1** (7.8-8.2)^*^	**7.7** (7.6-8.0)^*^
Body mass (kilograms)	19.4 (18.0-21.7)	19.7 (17.6-21.8)	21.6 (19.5-24.0)	21.7 (19.6-24.8)	24.8 (22.1-27.6)	24.2 (21.6-28.2)	**27.7** (24.6-32.9)^*^	**26.0** (23.3-30.4)^*^
Height (centimeters)	110.7 (108.5-114.2)	111.1 (107.5-114.4)	116.9 (114.0-120.6)	116.8 (113.1-120.5)	122.1 (118.4-126.8)	122.4 (118.6-126.5)	**129.0** (124.8-132.7)^*^	**126.4** (123.2-130.9)^*^
Body mass index (kg/m^2^)	15.9 (15.0-17.2)	15.9 (14.9-17.1)	15.6 (14.8-17.2)	16.0 (15.0-17.4)	16.4 (15.4-18.1)	16.2 (15.0-18.0)	**16.9** (15.4-19.1)^*^	**16.3** (15.3-18.1)^*^

^*^Significant differences (P < 0.05) between children who dropped out and those who continued to be followed up in the study. Values are expressed in medians and interquartile ranges. Groups were compared by means of the Mann-Whitney U test.

### Weight status classification

Body mass was assessed using a platform digital scale (model CA8000; G-life, São Paulo, Brazil), with precision of 0.1 kg. Height was measured using a portable stadiometer (Alturexata, Belo Horizonte, Brazil), with accuracy of 0.1 cm. The protocols followed the procedures described by Gordon et al.^18^ Body mass index (BMI) was determined from the body mass/height^2^ quotient, in which body mass was expressed in kilograms (kg) and height in meters (m).

The BMI cutoff points used followed the recommendations of the World Health Organization,^19^ in agreement with age and sex. Body mass index values were classified as underweight, normal weight, overweight or obese. For the purposes of this analysis, subjects classified as underweight and normal weight were grouped as normal weight (NW), while overweight (OW) and obesity (OB) were maintained as separate categories.

### Sports practice

Extracurricular sports practice was observed during data collection every year throughout the study, using the following dichotomous question “Do you participate in any extracurricular sports?” with answer options of “yes” or “no”. In the analysis in which extracurricular sports practice was tracked, the subjects were grouped into the following categories: practice to practice (P-P); practice to no practice (P-NP); no practice to practice (NP-P); or no practice to no practice (NP-NP).

### Statistical analysis

The sample were characterized in terms of medians and interquartile ranges. Data normality was tested using the Kolmogorov-Smirnov test, and comparisons were then made using the Wilcoxon test. BMI was tracked in relation to extracurricular sports practice and was evaluated through the percentage of subjects who remained in the same classification over the years. The agreement between baseline and follow-up was calculated by means of the kappa index, which takes into account the proportion of observed (Po) and expected (Pe) agreement (kappa: Po - Pe / 1 - Pe); analyses were performed stratified according to sex and group. Associations were analyzed using hazard ratios (HR) and assessed using the Cox multivariate regression model. All analyses were adjusted for sex, age, extracurricular sports practice and BMI at baseline. The software used was SPSS 25.0 (IBM, New York, United States) and values were considered significant at P < 0.05.

## RESULTS

Table 1 presents sample characterization values at both analysis times (baseline and follow-up). The sample consisted of 1,247 boys and 1,212 girls and all sample characterization values presented significant differences (P < 0.05) between the analysis times. Body mass and height values presented increases of approximately 30% and 15%, respectively, for both sexes by the end of the analysis period. These disproportionate increases resulted in higher body mass index values for this population at the time of the follow-up*.*

The tracked weight status values are presented in Table 2. It can be seen that 74.4% of the boys presented maintenance of weight status classification over the three years, with kappa classified as moderate (K = 0.523; P < 0.01), while 76.8% of the girls maintained this, with moderate kappa index (K = 0.537; P > 0.01). In contrast, approximately 25% of the subjects changed their weight status classification. This occurred mostly among individuals in the normal weight group, who increased their BMI. Only a small proportion of the overweight or obese children at the baseline changed their weight status to normal weight.

**Table 2.** Characteristics of the sample, separated according to sex, between the baseline and the follow-up

	Boys (n = 1,247)			Girls (n = 1,212)		
	Baseline	Follow-up	P	Baseline	Follow-up	P
Age (years)	6.4 (5.6-7.1)	9.7 (8.7-10.2)	< 0.01	6.2 (5.5-7.0)	9.5 (8.6-10.0)	< 0.01
Body mass (kilograms)	22.9 (20.3-26.4)	32.7 (28.4-40.2)	< 0.01	22.0 (19.6-25.7)	32.2 (27.2-39.3)	< 0.01
Height (centimeters)	119.3 (114.0-124.5)	137.1 (131.6-142.3)	< 0.01	117.5 (112.5-123.0)	136.0 (130.3-142.2)	< 0.01
Body mass index (kg/m^2^)	16.1 (15.1-17.5)	17.4 (15.8-20.4)	< 0.01	16.0 (14.9-17.6)	17.3 (15.6-20.0)	< 0.01

Values are presented as medians and interquartile ranges. The baseline and follow-up values were compared and differences were considered significant if P < 0.05.

Table 3 shows that the children tended to remain within the same sports practice classification, since tracking values of 76.5% and 84.2% were reported for the boys and girls, respectively. The incidence of extracurricular sports practice was extremely low (12.3%), and only 1.8% of the individuals maintained this practice over the three years. The kappa values for the boys did not show significant agreement, and the girls presented a poor kappa index (K = 0.108).

**Table 3.** Tracking of weight status and sports practice between the baseline and follow-up

**Weight status**		**Boys (n = 1,245)** **% (n)**	**Girls (n = 1,211)** **% (n)**	**Total (n = 2,456)** **% (n)**
**Baseline**	**Follow-up**			
NW	NW	53.8 (667)	59.1 (716)	56.3 (1383)
NW	OW	10.2 (127)	9.2 (112)	9.7 (239)
NW	OB	3.1 (38)	1.5 (18)	2.3 (56)
OW	NW	4.1 (51)	3.8 (46)	3.9 (97)
OW	OW	7.7 (96)	8.4 (102)	8.1 (198)
OW	OB	5.8 (72)	6.0 (73)	5.9 (145)
OB	NW	0.5 (6)	0.7 (9)	0.6 (15)
OB	OW	2.2 (28)	1.8 (22)	2.0 (50)
OB	OB	12.9 (160)	9.3 (113)	11.1 (273)
*% Tracking*		74.4	76.8	75.5
*Kappa*		0.523^*^	0.537^*^	0.530^*^
**Extracurricular sports**		**Boys (n = 1,241)** **% (n)**	**Girls (n = 1,203)** **% (n)**	**Total (n = 2,444)** **% (n)**
**Baseline**	**Follow-up**			
P	P	1.7 (21)	1.8 (22)	1.8 (43)
P	NP	8.3 (103)	5.3 (64)	6.8 (167)
NP	NP	75.8 (941)	82.5 (992)	79.1 (1993)
NP	P	14.2 (176)	10.4 (125)	12.3 (301)
*% Tracking*		76.5	84.2	80.9
*Kappa*		0.009	0.108^*^	0.054^#^

^*^Values with P < 0.001; ^#^Values with P < 0.05.

NW = normal weight; EW = excess weight; P = practice; NP = no practice.

**Figure 1.**
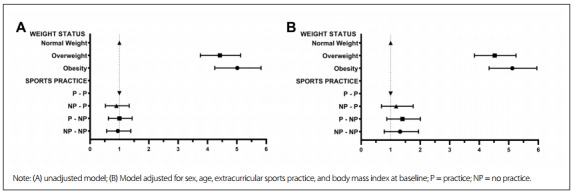
Cox regression model: hazard ratio of overweight or obesity in follow-up, with weight status at baseline and tracking of extracurricular sports practice.

Figure 1 presented the hazard ratio analysis (HR). This showed that overweight in childhood resulted in a 4.48 times higher risk of remaining in the same category or being classified as obese at the follow-up (odds ratio, OR: 4.48; confidence interval, CI: 3.83-5.24), compared with individuals of normal weight. Children classified as obese presented the same tendency, with a 5.08 times greater risk (OR: 5.08; CI: 4.33-5.95).

The tracking of extracurricular sports practice in relation to excess weight at the follow-up did not present significant results. The reference category was the group that practiced sports at both analysis times. For the group that practiced sports only at the time of the follow-up (NP - P), the OR was 1.10 (CI: 0.69-1.76). For the group that did not practice sports at the time of the follow-up (P - NP) or at either time (NP - NP), the ORs were 1.32 (CI: 0.87-2.00) and 1.23 (CI: 0.78-1.99), respectively.

## DISCUSSION

The aim of the present study was to ascertain the association between tracked extracurricular sports practice and weight status, and to track overweight and obesity among schoolchildren during three years of follow-up. The main finding was that children with excess weight maintained their classification after three years of evaluation. In addition, no association was found between tracked extracurricular sports practice and weight status. Only a low number of children participated in sports outside school.

Although no direct association was found between extracurricular sports practice and the prevalence of obesity at the time of the follow-up, it was observed that a large number of children did not practice sports frequently: approximately 85%. This is worrying, since sports practice may be directly associated with levels of physical activity and cardiorespiratory fitness during adolescence. Werneck et al.^20^ showed that sports practice was indirectly associated with metabolic risks and demonstrated that there was an association between sports practice during childhood and physical activity during adolescence.

In a systematic review, Hallal et al.^21^ confirmed the hypothesis that sports practice during childhood was directly linked to physical activity levels during adolescence. In addition, a study in Finland on 7,794 individuals found that sports practice during childhood led to greater likelihood that these children would become physically active adults.^22^ Moreover, maintenance of sports practice during adolescence is an essential indicator of weight status in adulthood; maintenance of sports practice during adolescence plays an important role in controlling BMI in adulthood.^23^

One possible explanation for the present results, in which no association was found between extracurricular sports practice and weight status, is that the sports practice tracked tended to be significant only for late adolescence and adulthood. Tracking of sports practice from early adolescence to adulthood shows that this practice seems to be not significant,^24^ thus suggesting that there is a specific point within adolescence (late adolescence) that influences individuals’ involvement in sports practice.

Moreover, sports practice has been correlated with daily calorie expenditure,^25^ which seems to be influenced by the intensity, duration, and frequency of sports. This is another possible factor affecting the association between sports practice and weight status, although the intensity, duration, and frequency of sports practiced were not evaluated in the present study.

Median body mass index values of 16.1 and 16.0 kg/m² at the baseline and 17.4 and 17.3 km/m² at the follow up were shown for boys and girls respectively in the present study. Over this three-year period, there was an increase in body mass index of 1.3 kg/m² for the boys and girls. The results from the present study are in accordance with findings from the NCD Risk Factor Collaboration, in which data on 128.9 million children and adolescents aged 5-19 years were analyzed. It was found that body mass index values were increasing by approximately 0.40 kg/m² every decade. These changes in body mass index had resulted in increased health risks relating to weight status.^26^

In a longitudinal analysis covering 22 years, Freedman et al.^27^ reported that high body mass index values in childhood were strongly associated with obesity in adulthood (BMI ≥ 40). Their results demonstrated that weight status had strong tendency to remain in the same classification from childhood to adolescence,^28^^,^^29^ and that the trend became stronger with increasing body mass index among children with severe obesity, in comparison with their peers.^27^ This can be explained, at least in part, by the fact that children who tend to maintain the same excess weight classification may have inadequate dietary habits and sedentary behavior. In other words, they consume larger amounts of sugary foods and spend more time watching television.^30^ However, these hypotheses could not be verified in the present study.

Likewise, a study in which body adiposity was tracked among children, based on skinfold measurements (subscapular and triceps) and controlled in relation to baseline body mass index, showed that 70% of the subjects remained in the highest tercile. It was also found that approximately 29% of the subjects classified in the low and medium terciles presented increased adiposity and were reclassified into the highest tercile over the three-year period.^10^ The results from that study were similar to those of the present study, in which 12.0% of the subjects changed from normal weight to overweight or obesity.

The results from the hazard ratio analysis on the children and adolescents at the three-year follow-up demonstrated that overweight or obese children at the baseline were 4.65 times (CI: 4.05-5.34) more likely to remain in the same classification or to move from the overweight group to the obese group. This was very similar to what was found in an American population that was followed up for 22 years, in which obese children were 2.7 times more likely to present class 3 obesity in adulthood.^27^

Obesity control is important, given that obese children are up to 1.5 times more likely to develop type 2 diabetes in adulthood than their peers with adequate weight status. A population that continually presented obesity from childhood to adulthood was three times more likely to develop type 2 diabetes.^31^ Children in the highest body mass index tercile were 4.8 and 1.9 times more likely to present altered fasting insulin and triglyceride values, respectively, than were their peers in the lowest tercile.^12^

Regarding limitations of the present study, some information relating to extracurricular sports practice was not obtained, including intensity, volume and type of sport practiced. Also, no information about sociodemographic variables was assessed. On the other hand, the strong points of the present study were that it provided a longitudinal analysis over a three-year period, with a large sample size in relation to the total population.

## CONCLUSION

The results from the present study demonstrated that there was a tendency for these children and pre-adolescents to present increases in body mass index values over time. Moreover, the risk that overweight children would gain weight (thus becoming obese) was higher than the risk that normal-weight children would gain weight. The analysis on extracurricular sports practice did not demonstrate any association with weight status among these schoolchildren. Further longitudinal studies should explore the characteristics of sports practice (intensity, volume, frequency and type), analyzed in relation to tracking of excess weight.
